# Editorial: Acoustic communication analysis for understanding animal behavior

**DOI:** 10.3389/fnbeh.2022.991573

**Published:** 2022-08-03

**Authors:** Sara Anna Bonini, Susanna Pietropaolo

**Affiliations:** ^1^Department of Molecular and Translational Medicine, University of Brescia, Brescia, Italy; ^2^Institut de Neurosciences Cognitives et Intégratives d'Aquitaine (INCIA), University of Bordeaux, CNRS, UMR 5287, Bordeaux, France

**Keywords:** acoustic communication, ultrasonic vocalizations, animal behavior, social context, communication analysis, automatic USVs analysis

Animals of most species emit vocalizations to communicate each other and to convey their emotional states. Intraspecific communication consists of the transfer of information from one or more animals [sender(s)] to one or more conspecifics [receiver(s)] in order to affect the current or future behaviors of the receiver(s). Vocal communication may occur both in the audible and ultrasonic frequency ranges and it is subjected by a complex brain modulation involving multiple areas and neural circuits. Communication is also impaired in a variety of pathological conditions, such as neurodevelopmental disorders (autism spectrum disorders, ASD, in particular), and these alterations can be tackled by pharmacological therapeutic strategies that have been proposed in several preclinical studies.

One of the most ambitious challenges of animal vocal communication concerns the study of ultrasonic vocalizations (USVs) in rodents and it consists of fully automatizing the procedures for spectrographic USV analysis. This would allow bypassing the operator-dependent and time-consuming analyses that are necessary for the quantitative and qualitative studies of USVs. This challenge was successfully faced by de Chaumont et al. in this special issue, as they developed one open-access software to automatically study mouse USVs. Their system also allowed coupling the spectrographic analysis of USVs with the automatic labeling of several behaviors scored over a long time-period (i.e., 3 days), thus providing an additional evaluation of the behavioral value of USVs in laboratory mice. The method was validated in C57BL/6J mice and also in mutants lacking the Shank3 gene, i.e., a mouse model of autism, in both sexes and at multiple ages. Also Goussha et al. developed a new software, the HybridMouse, that is an audio analysis tool for automatically identifying, labeling, and extracting recorded USVs. The methodological challenges of automatic processing a large amount of data from audio files does not concern only mouse ultrasonic communication. Here Marck et al. developed an automatic system based on audio signal processing algorithms and deep learning and applied to the vocal repertoire of the White Spectacled Bulbul (*Pycnonotus xanthopygos*), a bird species with a complex vocal communication system. Other modern computational methods for bioacoustics have been developed for the analysis of vocal communication in several animal species (including fish, amphibians, and insects), as described in detail in the review by Sainburg and Gentner.

Beside the analysis of the vocalizations emitted by the sender, the response of the receiving animal to vocalizations deserves to be studied. In rats, two main types of vocalizations are typically distinguished (Brudzynski, 2013; Wöhr and Schwarting, 2013): vocalizations with frequencies around 22 kHz, that are referred to as aversive or distress calls, presumably representing a negative affective state (Blanchard et al., 1991; Fendt et al., 2018), and vocalizations with frequencies around 50 kHz, that are thought to represent a positive affective state usually emitted during appetitive situations like play or mating (Knutson et al., 1998; Panksepp, 2005). In this context, Berz et al. investigated the emission of USVs in response to the playback of natural 50 kHz USVs in male juvenile rats of different strains. Their data demonstrated that most rats emitted response calls specifically linked to 50-kHz USV playback and these response calls were mostly characterized by a frequency range of 20–32 kHz and a mean duration of approximately 300 ms in all rat strains. The possible function of these type of calls has yet to be clarified, although it was unaffected by the pharmacological blockade of dopamine D2 receptors by haloperidol administration. In rats, the emission of USVs can be also promoted by operant conditioning during multiple weeks: this approach was employed in the study by Johnson et al. where a semi-automated method for training rats to increase their rate of USV production was introduced.

The impact of social context on animal communication is an issue of critical relevance that is far from being fully elucidated. Here, Warren et al. studied the ontogeny of prairie vole pup ultrasonic vocalizations when isolated or when the mother was present, but physically unattainable. They demonstrated a developmental maturation in all features of pup vocalizations and the impact of the different social contexts in modifying vocal emission. Capas-Peneda et al. reviewed instead the role of mouse USVs in the reproductive context; indeed, they summarized the most recent evidence demonstrating that USVs have an important role in parental cooperation, inducing both maternal and paternal behaviors. Bouguiyoud et al. investigated the effects of visual deprivation in acoustic communication and social behaviors using a mouse model of congenital blindness. They demonstrated here that congenital visual deprivation had no effect on the number of USVs emitted in pups and juveniles, but affected the USV emission in adult males and the social behaviors in juvenile and adult mice. The role of social isolation on adult USVs and social behaviors was instead assessed in both male and female mice by Premoli et al. Their study contributed to provide new guidelines for assessing ultrasonic communication in inbred mice, demonstrating also several sex differences in ultrasonic communication and social behaviors that were mostly unaffected by pre-testing social isolation.

Finally, another interesting aspect that has been investigated in a study included in this special issue concerns the molecular mechanisms underlying vocal communication. In particular, Aamodt and White examined whether microRNA-128 is behaviorally regulated in Area X (the striatopallidal song control nucleus) in juvenile zebra finches and found that its levels decline with singing. Furthermore, they demonstrated that inhibition of miR-128 in young birds enhanced the organization of learned vocal sequences, thus suggesting an important role for miR-128 in vocal communication and as a potential therapeutic target for autism spectrum disorders.

## Author contributions

All authors listed have made a substantial, direct, and intellectual contribution to the work and approved it for publication.

## Funding

SP received funding from Bordeaux University, CNRS, Association Autour de Williams and Fondation pour l'Audition (FPA-RD-2020-8). SAB received funding from the University of Brescia.

## Conflict of interest

The authors declare that the research was conducted in the absence of any commercial or financial relationships that could be construed as a potential conflict of interest.

## Publisher's note

All claims expressed in this article are solely those of the authors and do not necessarily represent those of their affiliated organizations, or those of the publisher, the editors and the reviewers. Any product that may be evaluated in this article, or claim that may be made by its manufacturer, is not guaranteed or endorsed by the publisher.
